# Cardiovascular Changes in Cirrhosis: Pathogenesis and Clinical Implications

**DOI:** 10.4103/1319-3767.65181

**Published:** 2010-07

**Authors:** Waleed K. Al-Hamoudi

**Affiliations:** Gastroenterology and Hepatology Unit, Department of Medicine, King Saud University, Riyadh, Saudi Arabia

**Keywords:** Cirrhosis, cirrhotic cardiomyopathy, hyperdynamic circulation, liver transplantation

## Abstract

Liver cirrhosis is associated with a wide range of cardiovascular abnormalities including hyperdynamic circulation, cirrhotic cardiomyopathy, and pulmonary vascular abnormalities. The pathogenic mechanisms of these cardiovascular changes are multifactorial and include neurohumoral and vascular dysregulations. Accumulating evidence suggests that cirrhosis-related cardiovascular abnormalities play a major role in the pathogenesis of multiple life-threatening complications including hepatorenal syndrome, ascites, spontaneous bacterial peritonitis, gastroesophageal varices, and hepatopulmonary syndrome. Treatment targeting the circulatory dysfunction in these patients may improve the short-term prognosis while awaiting liver transplantation. Careful fluid management in the immediate post-transplant period is extremely important to avoid cardiac-related complications. Liver transplantation results in correction of portal hypertension and reversal of all the pathophysiological mechanisms that lead to the cardiovascular abnormalities, resulting in restoration of a normal circulation. The following is a review of the pathogenesis and clinical implications of the cardiovascular changes in cirrhosis.

Patients with end-stage liver disease manifest a hyperdynamic circulation characterized by a decrease in the systemic vascular resistance and arterial pressure, and an increase in the heart rate and cardiac output. The clinical manifestations of hyperdynamic circulations include warm skin, spider angioma, palmer erythema, and bounding pulse. These cardiovascular changes were described over 50 years ago by Kowalski and Abelmann in a group of alcoholic cirrhotic patients.[1] These findings were then confirmed in multiple experimental models of portal hypertension and in patients with cirrhosis. Initially it was thought that these changes were a manifestation of latent alcoholic cardiomyopathy, however future studies confirmed the same circulatory dysregulation in cirrhotic patients with different underlying diseases.[2–4]

Different pathophysiological mechanisms including neurogenic, humoral, and vascular dysregulations are implicated in the pathogenesis of these cardiovascular changes. The hyperdynamic circulation is most likely initiated by splanchnic and peripheral vasodilatation, leading to reduction in the effective arterial blood volume. This leads to a diminished renal blood flow in cirrhotic patients, which in turn stimulates the rennin-angiotensin-aldesterone system (RAAS), sympathetic nervous system, and antidiuretic hormone resulting in renal artery vasoconstriction, sodium retention, and volume expansion.[5] Worsening liver disease results in progressive vasodilatation, making the hyperdynamic circulation and renal artery vasconstriction more pronounced.[6–7]

These circulatory changes lead to the development of multiple life-threatening complications including hepatorenal syndrome (HRS), ascites, spontaneous bacterial peritonitis (SBP), gastroesophageal varices, and hepatopulmonary syndrome.

The following is a review of the pathogenesis and clinical implications of the cardiovascular changes in cirrhosis.

## SYSTEMIC AND SPLANCHNIC CIRCULATION IN CIRRHOSIS

Arterial vasodilatation is regulated by complex interactions of different vasodilator molecules. Cirrhosis leads to portal hypertension which induces portosystemic collaterals, allowing gut-derived humoral substances to directly enter the systemic circulation without detoxification by the liver. In recent years, nitric oxide (NO) has been recognized as the most important vasodilator molecule in the splanchnic and systemic circulation. NO is overproduced in cirrhosis; measured serum levels are significantly elevated in both cirrhotic patients and in animal models.[8–10] NO is an endothelial-derived relaxing factor that leads to arterial vasodilatation. Its distribution differs in the splanchnic circulation; the intrahepatic microcirculation is altered significantly in liver cirrhosis, secondary to both architectural and vasoactive humoral changes, resulting in an increase in vasoactive molecules including angiotensin II, endothelin 1, and cysteinylleukotrienes associated with a decrease in intrahepatic NO production. The net result is a progressive increase in intrahepatic vascular resistance and subsequently portal hypertension. On the other hand, NO overproduction has been clearly documented in the remaining part of the splanchnic circulation in patients with cirrhosis. This is thought to be related to the altered intestinal mucosal permeability in cirrhotic patients, leading to the transfer of different endotoxins across the intestinal membrane to the systemic circulation. These endotoxins promote NO production.[11,12] Cytokines including TNF-α are among other factors that are considered to be NO inducers, whose effect has been demonstrated in a number of animal model studies.[13–15] Inhibition of TNF-α production or the blocking of TNF-α mediated signaling by TNF-α antibodies, resulted in normalization of NO production in experimental studies, which in turn leads to an improvement in the hyperdynamic circulation.[14,15]

Three isoforms of NO synthase (NOS) have been described: endothelial (eNOS), inducible (iNOS), and neuronal (nNOS). However, the leading isoform contributing to these vascular changes remains obscure. Multiple studies using nonspecific NOS inhibitors revealed that diminishing NO production resulted in normalization of the peripheral vascular vasodilatation.[16] Ferguson *et al* demonstrated that the selective inhibition of iNOS causes peripheral vasoconstriction in patients with cirrhosis but not in healthy-matched controls, suggesting the contribution of NOS in the regulation of peripheral vascular tone and the hyperdynamic circulation of end stage liver disease.[17] Other studies favor eNOS as the leading source of the vascular NO overproduction in the splanchnic arterial circulation.[18–19]

Other agents that are thought to play a role in the peripheral vasodilatation of cirrhosis include endocannabinoids.[20] They are lipid-like substances that act on two inhibitory G protein-coupled receptors, CB1 and CB2. CB1 receptors are upregulated in the vascular endothelium of cirrhotic rats in association with an increase in endocacannabinoids monocyte production. Activation of endothelial cannabinoid 1 (CB1) receptors by the endogenously produced endocannabinoids causes pronounced vasodilatation in the cirrhotic rats.[21]

More recently, multiple studies have shown a potentially important role of the central nervous system in the pathogenesis of the portal hypertension-induced hyperdynamic circulation.[22–25] Liu *et al*, demonstrated that primary afferent denervation by capsaicin, reversed the hyperdynamic circulation in cirrhotic rats. These findings suugest that primary afferent nerves may be the signaling pathway from the periphery to the CNS. Detection of a marker protein (Fos) in the brainstem and hypothalamic cardiovascular-regulatory nuclei of rats, immediately following portal vien ligation, indicates CNS activation. This is followed by hyperdynamic circulation, suggesting that the CNS activation plays a role in the pathogenesis of the hyperdynamic circulation. Additionally, blocking CNS Fos expression in cirrhotic rats resulted in eliminating the development of the hyperdynamic circulation.[25]

## THE HEART (CIRRHOTIC CARDIOMYOPATHY)

In addition to the hyperdynamic circulation, impaired ventricular contractility in response to stimuli was also described in cirrhotic patients.[26–27] Initially, this was thought to be a manifestation of latent alcoholic cardiomiopathy but later studies in non-alcoholic patients and in experimental animal models revealed the same pattern of blunted cardiac contractile responsiveness.[28,29] Thus, these cardiovascular changes are now termed “cirrhotic cardiomyopathy.”[3,4] Due to the associated systemic vasodilatation, overt heart failure is generally not a feature of cirrhotic cardiomyopathy.

In the absence of specific diagnostic criteria, the exact prevalence of cirrhotic cardiomyopathy remains unclear. The characteristic features of cirrhotic cardiomyopathy include (a) an attenuated systolic or diastolic response to stress stimuli, (b) structural or histological changes in cardiac chambers, (c) electrophysiological abnormalities, and (d) serum markers suggestive of cardiac stress.

The impaired cardiovascular responsiveness in cirrhosis is likely related to a combination of factors that include cardiomyocyte plasma membrane physicochemical changes, attenuated stimulatory pathways, and enhanced activity of inhibitory systems which includes the following:

1. β -adrenergic receptor function: The role of β-adrenergic receptors was examined in multiple studies. Heart cell contractility is mainly determined by the stimulatory β-adrenergic receptor system.[30] This system consists of the receptor, heterotrimeric guanine nucleotide-binding proteins (G-proteins), and adenylate cyclase. Catecholamine stimulation of the β-adrenoceptor leads to a number of interactions resulting in the production of the second messenger, cAMP. cAMP is the primary trigger that leads to intracellular calcium fluxes and cardiac muscle contraction.[31–32] Several studies examined this pathway in cirrhotic patients and animal models. β-adrenergic receptor density in cirrhotic patients and animal models were reduced.[28] Ma *et al*. found that β-adrenoceptors signaling pathway is also impaired at different levels, starting with decreased membrane content and function of the stimulatory Gs-protein and ending up with impaired activity of the adenylate cyclase enzyme itself.[33–34]
2. Muscarinic receptor activity: Muscarinic responsiveness was found to be blunted in cirrhotic hearts.[35] Muscarinic receptor stimulation exerts a negative inotropic effect on cardiac muscle counterbalancing the stimulatory β-adrenergic system. Thus, an enhanced muscarinic tone could contribute to the pathogenesis of negative inotropic effects on the myocardium.
3. Membrane fluidity: Membrane fluidity is a term used to describe the degree of motional freedom for lipid moieties in the lipid bilayer of the plasma membrane.[36] It has been demonstrated that the fluidity of the plasma membranes from heart cells and other tissues is decreased in cirrhotic patients, impairing the physiological function of the tissue biomembranes.[34,36] These changes have a profound effect on the β-adrenoceptor function that includes impairing the receptor-ligand interaction, receptor density, and intracellular signaling pathway. Restoration of normal values of membrane fluidity in an animal model resulted in a significant improvement in the function of β-adrenoceptors.[33]
  Altered membrane fluidity also affects the function of membrane-bound ion channels. Moreau *et al*. have shown altered control of vascular tone by Ca^++^ and K^+^ channels. In their study, potassium channel blockers resulted in a significant increase in the vascular tone in cirrhotic rats compared to normal rats.[37,38] Another study showed a decrease in the function of two types of K^+^ channels in ventricular myocytes from cirrhotic rats, which could potentially explain the prolongation of the QT interval.[39] Additionally, the movement of calcium into the cell is altered affecting cardiomyocyte contraction. Calcium enters the cell through membrane calcium channels, and is stored and released from the sarcoplasmic reticulum intracellular stores. The calcium channel dysfunction leading to decreased cardiomyocyte contractility was also demonstrated in an animal model study.
4. Nitric oxide: NO plays an important role in regulating the systemic and coronary vascular tone and its effect has been implicated in the pathogenesis of different cardiac dysfunctions including ischemic heart disease.[40,41] Non-selective blockade of nitric oxide synthase (NOS) by N omega-monomethyl-L-arginine (L-NMMA) augments the contractile response of rat ventricular myocytes to the β-agonist isoproterenol without any effect on the baseline contractility, suggesting the inhibitory effect of NO on cardiac contractility.[42] Similarly, in another study, inhibition of NO synthesis with L-NMMA restored the blunted contractility function of isolated hearts from cirrhotic rats, while it had no significant effect on the animals in the control group.[43]
5. Carbon monoxide (CO): CO is a known vasodilator that also exerts a negative effect on cardiac contractility through cGMP.[44] CO stimulates guanylate cyclase to produce cyclic guanosine monophosphate (cGMP), which phosphorylates protein kinase G and inhibits calcium influx into the cytosol of the cardiomyocyte. Lui *et al*. investigated its role in the pathogenesis of cirrhotic cardiomyopathy. They showed that HO-1 mRNA transcription and protein expression are significantly augmented in ventricles of cirrhotic animals compared to controls. They also showed that the hemeoxygenase inhibition significantly decreased the elevated cGMP content and reversed the decreased contractility of isolated BDL papillary muscles with no affect on control muscles.[45] Based on these findings, they suggested that activation of the HO/CO pathway is involved in the pathogenesis of cirrhotic cardiomyopathy.
6. Endocannabinoids: Upregulation of the cannabinoid signaling pathway has been described in chronic liver disease.[21] Endogenous cannabinoids exert a negative inotropic effect in human and animal models through their interaction with the inhibitory G-protein-coupled receptors, CBI and CB2, leading to the inhibition of adenylate cyclase activity and calcium influx into the cytosol of the cardiomyocyte.[46,47] Gaskari *et al*. showed that the blunted contractile response of isolated left ventricular papillary muscle from cirrhotic rats is restored after preincubation with a CB1 antagonist.[48]

### Clinical implications

This circulatory dysfunction characterized by arterial vasodilatation and an increase in cardiac output is the proposed pathophysiological mechanism leading to sodium and water retention in patients with liver cirrhosis.[49] Initially the decrease in systemic vascular resistance is compensated by the development of a hyperdynamic circulation. However, with disease progression, the extreme extension of these hemodynamic changes leads to severe renal vasoconstriction and a decline in renal function which characterizes the HRS.[50,51] The development of hepatorenal syndrome in this setting is also associated with a drop in the cardiac output, emphasizing the additional role of cirrhotic cardiomyopathy in the pathogenesis of hepatorenal dysfunction. A recent study compared the hemodynamic changes in relation to sodium intake of 22 mmol/day between 35 cirrhotic patients, with or without ascites, and a group of healthy controls. After subjecting 14 patients with non-ascitic cirrhosis and 8 controls to a sodium load of 200 mmol/day for 7 days, they showed that patients with non-ascitic cirrhosis had cardiac contractile dysfunction which was augmented by the dietary sodium load and was also associated with significant sodium retention compared to the healthy controls.[52] Another study assessed the relationship between the development of HRS and cardiac dysfunction in a cohort of patients with SBP. They They demonstrated that patients who developed acute renal failure had a significantly less cardiac reserve, manifested by a lower cardiac output and a higher systemic vascular resistance at admission and following the treatment of the spontaneous bacterial peritonitis, when compared to patients who did not develop renal failure.[53–54]

Several indicators of poor prognosis in liver cirrhosis are those which occur in association with extreme peripheral arterial vasodilatation, including a low mean arterial blood pressure, portal hypertension, and the elevation of different neuroendocrinal substances. Thus, treatment targeting the circulatory dysfunction in these patients may improve the short-term prognosis while awaiting liver transplantation.[55–57] A recent study conducted by Esrailian *et al*. revealed that octerotide and midodrine therapy significantly improved renal function and 30 day survival in patients with type 1 hepatorenal syndrome.[58]

Transjugular intrahepatic portosystemic shunt (TIPS) insertion in cirrhotic patients leads to significant hemodynamic changes. TIPS causes a sudden increase in the preload in patients who are manifesting a hyperdynamic circulation at the time of the shunt insertion.[59–62] Multiple cardiac-related complications including arrhythmias, heart failure, myocardial ischemia, and acute pulmonary edema have been reported following TIPS insertion.[63] An increase in the right atrial pressure, pulmonary artery pressure, pulmonary vascular resistance, and pulmonary wedge pressure has been reported, reflecting the common prevalence of diastolic dysfunction in this patient population.[60,63] Predicting patients that are at a higher risk of developing cardiopulmonary complications after TIPS insertion remains difficult. A recent study assessed the utility of E/A ratio, an indicator of diastolic dysfunction, to predict ascites clearance and mortality after TIPS insertion. They found that an E/A ratio of ≤1 was predictive of slow ascites clearance (hazard ratio=7.3, 95% confidence interval=1.3-40.7, P=0.021) and death after TIPS (hazard ratio=4.7, 95% confidence interval=1.1-20.2, *P*=0.035).[64] These findings are in agreement with a previous study showing a higher mortality rate in patients with an E/A ratio of < 1.[65] These studies clearly demonstrate that sudden hemodynamic changes following TIPS insertion may not be tolerated by the already compromised cardiomyocytes, this emphasizes the importance of careful selection of patients prior to TIPS insertion.

The consequences of cirrhosis-related cardiovascular alterations can also be manifested at the time of transplantation. During orthotopic liver transplantation (OLT), the hyperdynamic circulation is further compromised by the effect of anesthesia, mechanical ventilation, and surgical clamping. This results in significant reduction in the preload and subsequently a reduction in the cardiac output.[66,67] Ripoll and colleagues investigated the cardiac response during liver transplantation. In their study they demonstrated that almost 25% of patients who undergo liver transplantation, show an abnormal cardiac response during the surgical procedure after reperfusion.[68]

Postoperatively, inappropriate fluid management could lead to either hypovolemia or fluid overload, which can also be a strain on the heart. Metabolic derangements in the immediate postoperative period can also impair the cardiac contractility and hence result in significant swings in the systemic hemodynamic parameters.

Hemodynamic depression caused by hypocalcemia-induced citrate intoxication from massive transfusion was reversed after the administration of CaCl_2_.[69] On the other hand, the systemic vasodilatation in cirrhotic patients is thought to play a protective role in preventing the development of heart failure in the setting of cirrhotic cardiomyopathy.

Cardiac complications following liver transplantation are common involving up to 70% of patients; fortunately most of these complications are subclinical.[70–72] Pulmonary edema is the most common cardiovascular complication with at least 50% of these episodes developing immediately after transplantation.[67,73] Post-operative mortality from heart failure and cardiyomyopathy has been reported shortly after transplantation.[66,67] Careful fluid management in the immediate post-transplant period is extremely important to avoid cardiac-related complications. Post-operative myocardial depression, poor cardiac output, hypoxemia and mortality were associated with poor preoperative cardiac reserve.[74–76] Predicting the development of postoperative cardiac complications is very difficult. Two-dimensional and dobutamine stress echocardiography were utilized to predict the development of adverse cardiac events following liver transplantation and both had a low predictive value.[73] In a recent study, Fouad *et al*. reviewed one hundred and ninety-seven patients to clarify the factors that may be able to predict cardiac complications following OLT, of whom 40% suffered one or more cardiac complications within 6 months of the surgery. Pulmonary edema was the most common complication occurring in 30% of patients; other complications included overt heart failure, arrhythmia, pulmonary hypertension, pericardial effusion, and cardiac thrombus formation. In this study, adverse intraoperative cardiovascular events, previous cardiac disease, and advanced liver disease as quantified by MELD score, predicted post-operative cardiac complications. Other variables including age, sex, OLT indication, body mass index, blood pressure and smoking, had a poor predictive value. Additionally, none of the pre-OLT investigations including chest X-ray, electrocardiogram, echocardiography, coronary angiopgraphy, pulmonary arterial pressure and 2-methoxy isobutyl isonitrile scan, predicted post-operative cardiac complications.[67]

## THE PULMONARY CIRCULATION

Intrapulmonary vascular abnormalities consisting of pulmonary vascular dilatation, intrapulmonary shunting and a low pulmonary vascular resistance, have been described in patients with liver cirrhosis[77,78] This is thought to be related to the effect of multiple vasoactive substances including NO. Measured NO level increases in patients with cirrhosis and normalizes after liver transplantation.[79–80] Pulmonary alveoli are another source of NO production in cirrhotic patients. An increase occurs in both iNOS and eNOS isoforms in alveolar macrophages and pulmonary endothelial cells of cirrhotic patients.[81,82] NO inhibition resulted in a transient improvement in the pulmonary vascular disturbance.[83,84] Other studies did not confirm the same findings, suggesting that other factors may play a significant role in the pulmonary vascular tone.[85] Despite the similar effect of these vasoactive substances on both the systemic and pulmonary circulation, other pulmonary specific related mechanisms may play a major role. Hepatopulmonary syndrome, which is one of the end results of these vascular changes, can happen in early cirrhosis, in which systemic hemodynamics are usually normal.[86,87] Additionally, hepatopulmonary syndrome is only reported in around 30% of patients with advanced cirrhosis.

Endotoxin, TNF-α, endothelin-1 (ET-1), and CO are among other factors that may contribute to the pulmonary vascular changes.[88] More recently, studies focused on the role of intravascular macrophages on the pulmonary hemodynamics. Phagocytically active pulmonary intravascular macrophages accumulate in the lungs after common bile duct ligation (CBDL) in an animal model.[8] Carter *et al*. demonstrated in a CBDL model that not only does the macrophage production of of NO increase, but it also has an up-regulating effect on the CO production, contributing further to the pulmonary hemodynamic changes.[9] Circulating TNF-α appears to have a significant role in the accumulation of intravascular macrophages in CBDL animal models; inhibition of TNF-α results in a decrease in the number of macrophages and improves the hepatopulmonary syndrome.[10,89] Intrapulmonary and portopulmonary shunts are among other clearly described changes in patients with liver cirrhosis that have significant clinical implications.[90,91]

Portopulmonary hypertension is another syndrome that has been associated with end-stage liver disease. Portopulmonary hypertension is less prevalent than hepatopulmonary syndrome, with an estimated prevalence of around 5%.[92,93] The pathogenesis of this syndrome remains unclear. A group of investigators believe that vasoactive substances that are normally metabolized in the healthy liver, escape the detoxification and are allowed to reach the pulmonary circulation and account for the increased pulmonary pressure.[94,95] Others believe that the portopulmonary hypertension results from high cardiac output, exposing the pulmonary vasculature to increased shear stress. As a result, some patients may respond with vasoconstriction, hypertrophy and proliferation of the pulmonary arterial endothelial cells, leading to portopulmonary hypertension.[96] A third group of investigators suggested that portopulmonary hypertension results from venous thromboembolism arising from blood clots passing through portosystemic shunts before reaching the pulmonary circulation.[97] Finally, certain genetic mutations have been linked to the development of pulmonary hypertension in different diseases.[98,99] Whether a specific genetic defect plays a role in the pathogenesis of portopulmonary hypertension remains unclear.

### Clinical implications

The pulmonary vascular dilatation and the intrapulmonary shunts in patients with liver cirrhosis are the leading contributors to hypoxemia in advanced liver disease. The low pulmonary vascular resistance leads to a reduction in the intrapulmonary transit time. This leads to a decrease in the oxygen diffusion across the dilated pulmonary vessels, and has been labeled as the alveolar-capillary disequilibrium hypothesis.[100] Based on the above hypothesis, there is no true shunts and the associated hypoxemia can be improved by oxygen supplementation. Another pattern of intrapulmonary vascular dilatation, characterized by localized dilatation of parts of the pulmonary vasculature and associated with large arteriovenous shunting distant from the gas exchange units has been described. This type has a true anatomical shunting, therefore the response to oxygen supplementation is poor.[101,102] The presence of numerous intrapulmonary shunts was first described by Hoffbauer *et al*, following which multiple imaging studies using various molecules confirmed these findings.[103–105] Despite the relatively high prevalence of hypoxemia in cirrhotic patients, it is uncommon for these patients to die of respiratory failure. However, one study compared the median and 5 year survival in two groups with and without hepatopulmonary syndrome. The two groups were matched for age, etiology, and severity of liver disease according to the Child classification and the MELD score. The patients with the hepatopulmonary syndrome had a worse median and 5 year survival.[106]

Liver transplantation is the only definitive treatment for hepatopulmonary syndrome with at least 85% of patients experiencing significant improvement or complete resolution of hypoxemia following surgery.[107] However, patients with hepatopulmonary syndrome have a higher post-transplant mortality rate when compared to patients who do not have the syndrome.[108] Previously reported post-operative complications in patients with hepatopulmonary syndrome include pulmonary hypertension, cerebral embolic hemorrhages, and prolonged mechanical ventilation.[109–110]

Portopulmonary hypertension is associated with high mortality, ranging between 50-90% in 5 years. Kawut *et al*. compared survival in patients with potopulmonary hypertension versus idiopathic pulmonary hypertension and showed that patients with portopulmonary hypertension have a higher risk of death when compared to patients with idiopathic pulmonary hypertension.[111] Postoperative mortality is usually higher in patients with a lower cardiac reserve preoperatively. As opposed to hepatopulmonary syndrome, liver transplantation is contraindicated in patients with severe portopulmonary hypertension. Recent data suggest a perioperative mortality rate of around 70% in patients with mean pulmonary artery pressure >45 mmHg.[112,113] Patients with less severe portopulmonary hypertension may benefit vasodilator therapy to reduce the pulmonary pressures prior to transplantation.[114]

## LIVER TRANSPLANTATION

Liver transplantation results in correction of portal hypertension and reversal of all the pathophysiological mechanisms that lead to hyperdynamic circulation. The effect of liver transplantation on the systemic hemodynamics has been studied by different groups. We studied the hemodynamic changes of 66 patients in the immediate postoperative period, comparing patients with viral and alcoholic cirrhosis.[115] We found that within the first 24-h of transplant, there was a significant decrease in the heart rate (HR) and an increase in the mean arterial pressure (MAP), the extent of change was similar in the two groups. The central venous and pulmonary capillary wedge pressures, and systemic vascular resistance increased, and cardiac index decreased in the viral but not the alcoholic patients. Patients with alcoholic cirrhosis showed a lower pulmonary vascular resistance and pulmonary artery pressure compared to the viral group at 24 h. Although the hyperdynamic circulation persists in the immediate post-transplant period, systemic parameters improve faster in the viral group. We also showed that pulmonary hemodynamics differ significantly between the two groups within the first 24 h, suggesting that alcoholics may have more pronounced pulmonary vasodilation than viral-cirrhotic patients. Glauser studied the systemic hemodynamics within the first 96 h following liver transplantation in 21 patients; his results suggested a progressive improvement towards normality within the study period.[116] Navasa *et al*. examined the hemodynamics of 12 patients at 2 weeks and 2 months following liver transplantation and suggested that most of the hemodynamic changes reverse.[117] On the other hand, other studies showed persistence of the hyperdynamic circulation for at least 6 months post transplantation.[118,119] Henderson *et al*. studied the cardiac output in 21 patients before and at 1 or 2 years following transplantation. They showed that the high cardiac output persist at both time periods.[118] Another study showed that the hyperdynamic circulation persists in the first 6 months following transplantation, but at 12 months the hyperdynamic circulation improves significantly.[119] The authors of these studies suggest that persistent collateral circulation may be the reason for the slow but gradual improvement of the hyperdynamic circulation. The use of different diagnostic modalities and the limited number of patients in these studies, makes it difficult to draw definite conclusions on the exact effect of transplantation on the hemodynamics.
